# CO-DESIGNING A HIGH-ACCURACY HOME MONITORING SYSTEM FOR MANAGING FRAILTY IN OLDER ADULTS

**DOI:** 10.1093/geroni/igad104.3231

**Published:** 2023-12-21

**Authors:** Adriana Ríos Rincón, Andrew Chan, Mathieu Figeys, Farnaz Koubasi, Yusuf Ahmed, Geoffrey Gregson, Antonio Miguel-Cruz

**Affiliations:** University of Alberta, Edmonton, Alberta, Canada; Glenrose Rehabilitation Hospital, Alberta Health Services, Edmonton, Alberta, Canada; University of Alberta, Edmonton, Alberta, Canada; University of Alberta, Edmonton, Alberta, Canada; University of Ilorin, Ilorin, Kwara, Nigeria; Glenrose Rehabilitation Hospital, Alberta Health Services, Edmonton, Alberta, Canada; University of Alberta, Edmonton, Alberta, Canada

## Abstract

**Background:**

Frailty, a condition often affecting older adults, increases vulnerability and diminishes physical abilities across bodily systems. Current non-routine frailty screening in primary care or clinical settings fails to detect “hidden health vulnerabilities” in a timely manner. Smart home technologies offer an affordable and effective solution for continuous frailty tracking and prevention. However, existing home monitoring technologies typically require users to acquire new skills or are invasive, such as camera-based systems. Objective: Our goal is to create a high-accuracy home monitoring system coupled with Internet of Things devices to identify potential frailty indicators.

**Methods:**

This qualitative description study involves 4 to 8 participants, including older adults with and without Mild Cognitive Impairments/frailty, caregivers, and clinicians, in a group interview. Using card sorting and task mapping, the interview seeks to identify and define the features and challenges of a smart home system to monitor frailty in older adults.

**Results:**

The study is registered in clinical trials, with data collection commencing soon. At the conference, we will present the research protocol and the findings from the analysis of the interviews.

**Conclusion:**

Through the early engagement of older adults and caregivers, we strive to design a valuable and meaningful system that (1) uses zero-effort technologies so frail older adults do not need to develop new skills in order to use the system; (2) is a non-camera-based tracking technology preserving autonomy and privacy; (3) generates the frailty data meaningful for older adults, caregivers and the health system.

